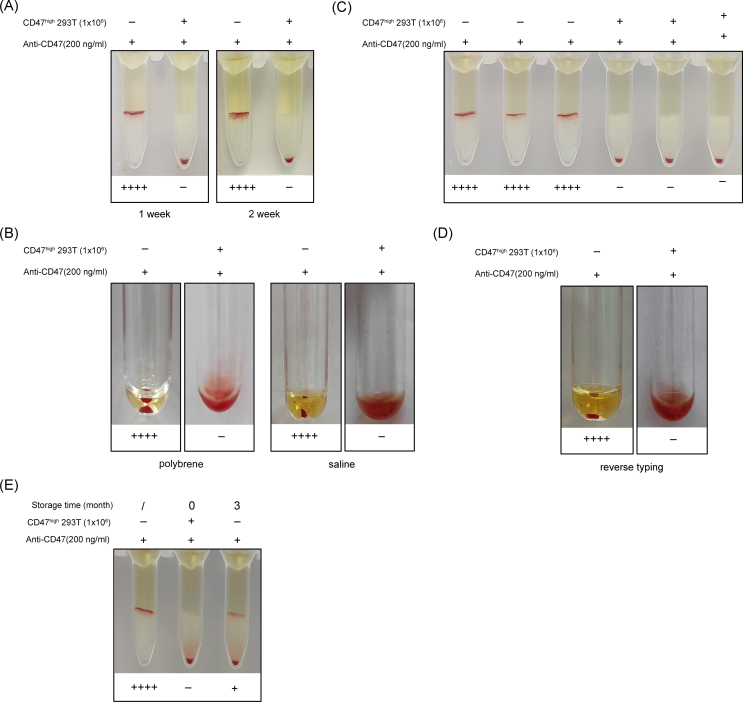# Depletion of anti-CD47mAb in plasma by genetically modified cells for pre-transfusion testing

**DOI:** 10.1016/j.gendis.2023.101104

**Published:** 2023-09-13

**Authors:** Fei Wang, Wenting Wang, Xiaoshuang Wu, Zhixin Liu, Yafen Wang, Rong Zhang, Shunli Gu, Qunxing An, Yaozhen Chen, Xingbin Hu

**Affiliations:** a Department of Transfusion Medicine, Xijing Hospital, Fourth Military Medical University, Xi'an, Shaanxi 710032, China; b Xi'an Medical University, Xi'an, Shaanxi 710021, China

Allogeneic red blood cell (RBC) transfusion is commonly performed in medical practice because of its efficacy and low-risk level. However, pre-transfusion tests are susceptible to monoclonal antibody (mAb) interference.^1^ Currently, mAb therapies are being developed to treat many diseases, such as cancer. However, certain mAbs, such as anti-CD38mAb and anti-CD47mAb, can bind to RBC membranes; this binding interferes with pre-transfusion tests.^2^ CD47 has gained considerable attention in recent years because of its potential as a therapeutic target for hematologic malignancies and solid tumors.^3^ The binding of anti-CD47mAb to RBCs may lead to false-positive results in pan-agglutination tests and cause delays and risks in establishing compatible RBCs for transfusion.

Gamma-clone anti-IgG can nullify the interference of anti-CD47mAb in pre-transfusion testing^2^; however, this method is costly and only suitable for the IgG4 subtype of anti-CD47mAb. Previously, expired RBCs or platelets in storage have been used to remove antibodies from patients.^4^ However, this method is inefficient and may cause depletion of other significant blood group antibodies. Thus, novel approaches are urgently needed to eliminate the interference of anti-CD47mAb. In this study, we designed human embryonic kidney (HEK)-293T cells expressing high levels of CD47 to adsorb anti-CD47mAb.

To test for the interference in pre-transfusion testing, we established a model by adding anti-CD47mAb to normal plasma (Fig. S1). Although human erythroleukemia (HEL) cells expressed CD47, they could not sufficiently deplete anti-CD47mAb at a concentration of 10 ng/mL (Fig. S2). To achieve satisfactory depletion efficiency, CD47^high^ 293T cells were produced using a lentiviral delivery system (Fig. 1A; Fig. S3). Adsorption of CD47^high^ 293T cells was performed to test their efficacy (Fig. 1B). Notably, 1.0 × 10^6^ CD47^high^ 293T cells absorbed 200 ng/mL anti-CD47mAb, decreasing abnormal agglutination from 4+ to 0, demonstrating that these cells were more effective than HEL cells (Fig. 1C, D). Interference from anti-CD47mAb in the polybrene and saline tests, irregular antibody screening, and reverse blood group typing, was also eliminated by CD47^high^ 293T cells (Fig. 1E–G). These results indicate that CD47^high^ 293T cells depleted anti-CD47mAb more effectively than the HEL cell line.

**Figure 1:**
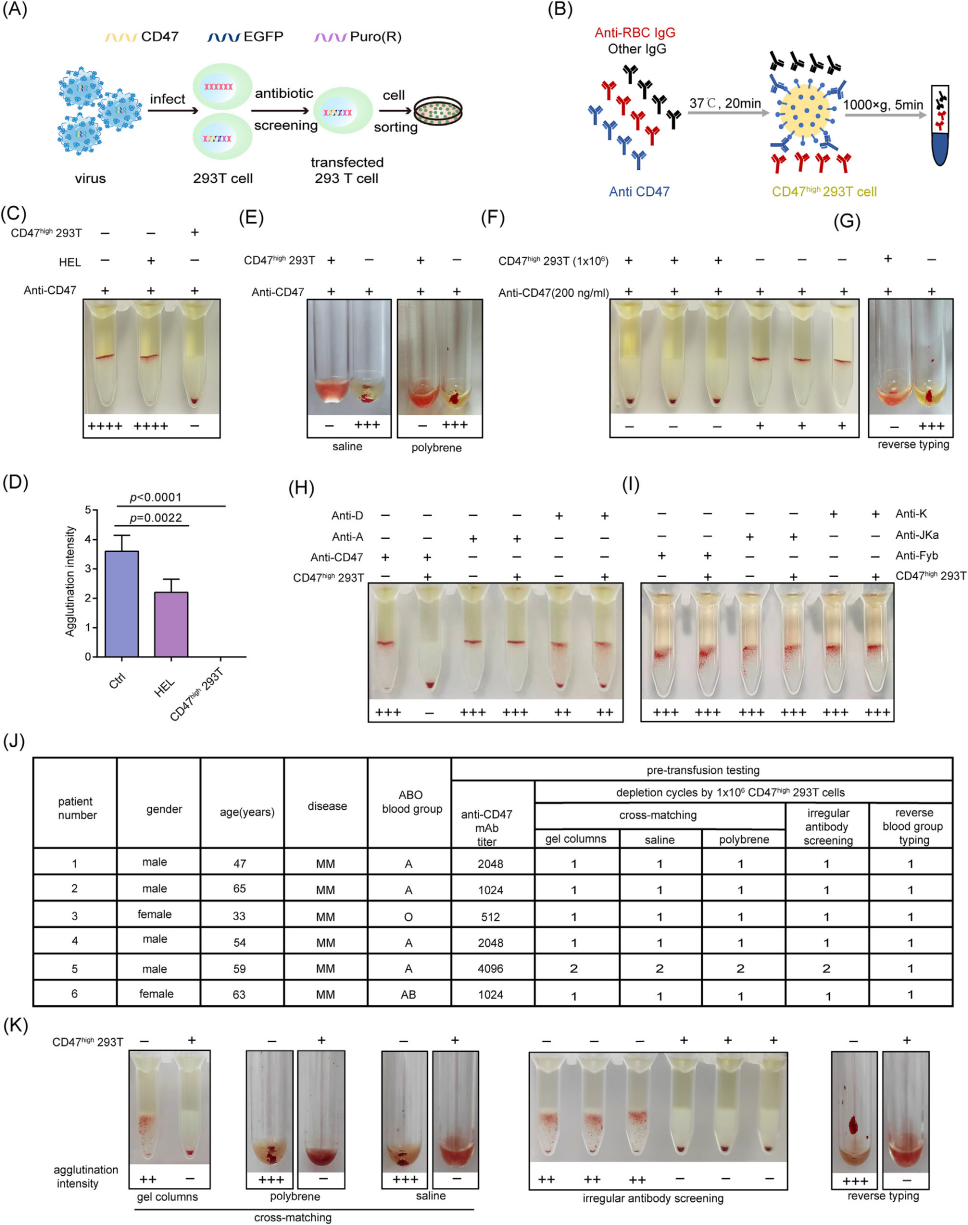
CD47^high^ 293T cells depleted plasma anti-CD47mAb in pre-transfusion testing. **(A)** Preparation of HEK-293T cells expressing high levels of CD47 through lentiviral transfection. **(B)** Depletion of anti-CD47mAb by CD47^high^ 293T cells in plasma. **(C)** Agglutination intensities, according to the gel column method, were detected after treatment with 1.0 × 10^6^ HEL or 1.0 × 10^6^ CD47^high^ 293T cells. **(D)** Depletion efficiency of HEL and CD47^high^ 293T cells. **(E)** Detection of interference in saline and polybrene-mediated cross-matching after treatment with CD47^high^ 293T cells. **(F)** Detection of interference in irregular antibody screening using the gel column method after treatment with CD47^high^ 293T cells. **(G)** Blood group B was reverse-typed with the saline method after treatment with CD47^high^ 293T cells. **(H)** Plasma samples for B or RhD + typing were incubated with CD47^high^ 293T cells, and the cells were removed via centrifugation. The agglutination intensities were detected using the gel column method. **(I)** Anti-K, anti-Fyb, and anti-JKa were mixed with plasma. **(J)** Samples from six patients were processed with CD47^high^ 293T cells and analyzed through pre-transfusion testing. Information about the six patients, anti-CD47mAb plasma sample titers, and depletion cycles with CD47^high^ 293T cells for removing interference in pre-transfusion testing. **(K)** Photographs of pre-transfusion testing of one representative patient, including cross-matching, irregular antibody screening, and reverse-typing. Agglutination was scored on a scale of 0–4+.

To evaluate whether CD47^high^ 293T cells can be used in clinical practice, we fixed CD47^high^ 293T cells using 4% paraformaldehyde and stored them at 4 °C for two weeks. Thereafter, we examined their ability to deplete anti-CD47mAb. The results indicate that stored CD47^high^ 293T cells maintained their depletion capability (Fig. S4A–D). Even after three months of storage, CD47^high^ 293T cells still retained their adsorption capacity (Fig. S4E). Thus, we developed a feasible strategy for absorbing anti-CD47mAb from stimulated plasma in pre-transfusion tests.

Whether a method affects other antibodies relating to blood group reactions is a practical concern. To address this issue, we incubated anti-B or anti-D antibody-positive plasma with CD47^high^ 293T cells and then removed the cells through centrifugation. The results revealed no difference in the agglutination of anti-A and anti-RhD antibodies with RBCs after treatment with CD47^high^ 293T cells (Fig. 1H). Similarly, we mixed anti-Fyb, anti-JKa, and anti-K mAbs with CD47^high^ 293T cells in the plasma and found that agglutination was not altered (Fig. 1I). Therefore, CD47^high^ 293T cells avoid absorbing other blood group antibodies.

As CD47^high^ 293T cells efficiently depleted anti-CD47mAb *in vitro*, we further explored their application in clinical patient samples. Six plasma samples from patients who were recently administered lemzoparlimab were used in titration experiments. The anti-CD47mAb titers in the plasma samples were 512–4096 (Fig. 1J). After CD47^high^ 293T cell treatment, anti-CD47mAb interference in gel column-mediated cross-matching was depleted (Fig. 1K). Abnormal reactions in polybrene and saline-mediated cross-match tests were also prevented by CD47^high^ 293T cells (Fig. 1K). Anti-CD47mAb interference in irregular antibody screening tests and reverse blood group typing was also ruled out after cell-based depletion (Fig. 1K). Five plasma samples were reproducibly cleared of free pan-agglutinin following a single depletion step with CD47^high^ 293T cells; only one sample required a second depletion cycle (Fig. 1K). Thus, samples with titers up to 2048 were reliably eliminated with one depletion. The need for a second depletion cycle was restricted to the sample with a titer > 2048. Thus, we established a method that avoids anti-CD47mAb perturbation during transfusion services. Our proposed method has several advantages. HEK-293T cell culture is simple, and these cells are commonly used in bio-engineering. Depletion of anti-CD47mAb with 1.0 × 10^6^ CD47^high^ 293T cells was achieved, particularly compared with a previous method that requires 3.0 × 10^7^ Darasorb cells to absorb anti-CD38mAb.^5^ Furthermore, our depletion method is effective even at high anti-CD47mAb concentrations. We also achieved anti-CD47mAb depletion in polybrene and saline medium tests.

In conclusion, we developed a strategy for depleting anti-CD47mAb to eliminate interference in pre-transfusion testing. This strategy can help transfusion services to cope with anti-CD47mAb administration in cancer therapy. With the development of a standard protocol and effective laboratory management, bio-engineering cell-based depletion of mAbs more than anti-CD47mAb will benefit patients who require RBC transfusions.

## Ethics declaration

All human blood samples were collected after obtaining informed consent from blood donors and patients. The experimental procedures were performed in accordance with the requirements of the Xijing Hospital Ethics Committee.

## Author contributions

XBH, YZC, and QXA designed the study and conducted experiments. FW, WTW, XSW, RZ, and SLG carried out the experiments and analyzed the results. ZXL and YFW critically evaluated the data. XBH and YZC wrote the manuscript, which was additionally edited and commented on by the others.

## Conflict of interests

The authors declare no competing interests.

## Figures and Tables

**figs1:**
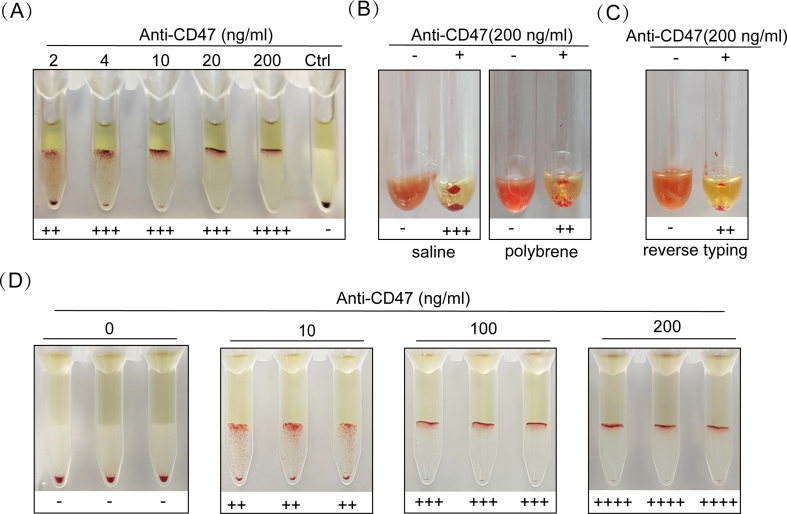


**figs2:**
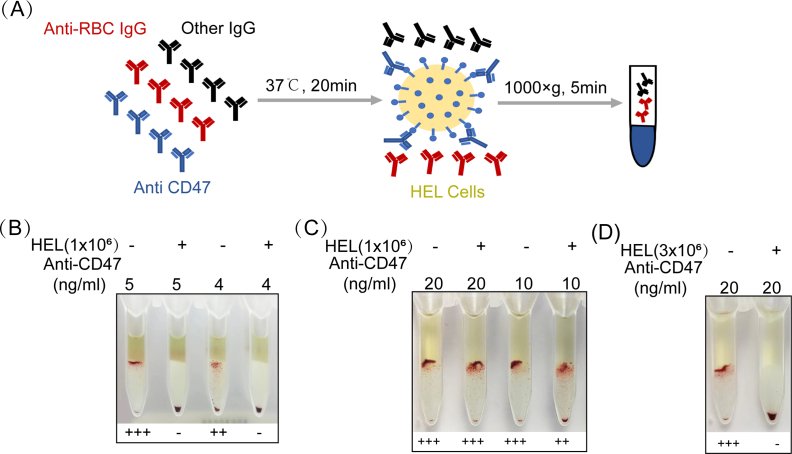


**figs3:**
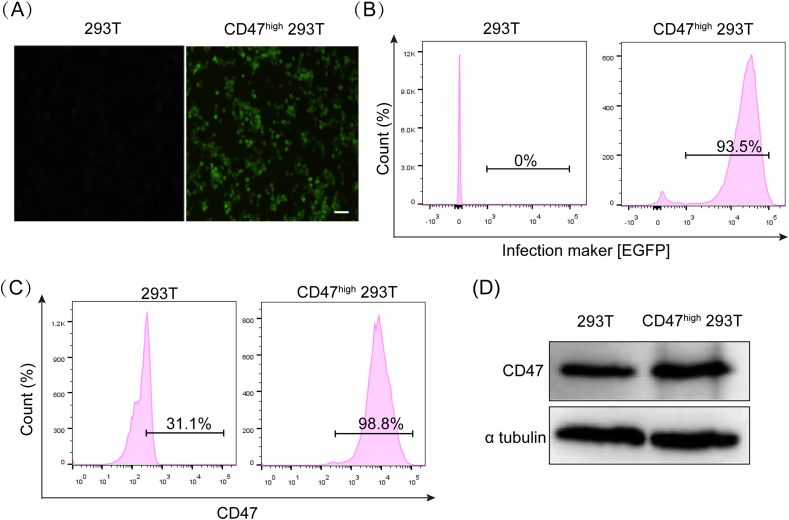


**figs4:**