# Supplementary material for: Depletion of anti-CD47mAb in plasma by genetically modified cells for pre-transfusion testing
Source: Genes Dis. 2023 Sep 13;11(5):101104. doi: 10.1016/j.gendis.2023.101104 (PMC11176643; doi:10.1016/j.gendis.2023.101104)
Supplement: Multimedia component 1 [file mmc1.docx]

Supplemental Materials

Depletion of anti-CD47mAb in plasma by genetically modified cells for pre-transfusion testing

Fei Wang^1,2,#^, Wenting Wang^1, #^, Xiaoshuang Wu^1, #^, Zhixin Liu^1^, Yafen Wang^1^, Rong Zhang^1^, Shunli Gu^1^, Qunxing An^1,*^, Yaozhen Chen^1,*^, Xingbin Hu^1,*^

Correspondence Authors**, ^#^** They equally contributed to this study.

Email addresses: [hxbyqh@163.com](mailto:hxbyqh@163.com) (X.Hu), [zhenzhenscu@126.com](mailto:zhenzhenscu@126.com) (Y. Chen), [bestar01@163.com](mailto:bestar01@163.com) (Q.An)

**Supplemental materials and methods**

**Cell and blood samples:** Human erythroleukemia (HEL) and human embryonic kidney (HEK)-293T cells were purchased from BLUEFBIO Company, Shanghai, China. Blood samples were obtained from donors. In total, six plasma samples were collected from patients with multiple myeloma, and patients were administered lemzoparlimab (I-MAB Biopharma, Shanghai, China).

**Reagents:** For anti-CD47mAbs, we used anti-CD47 (#CC2C6, Biolegend, San Diego, CA, USA) and anti-CD 47 (#b6h12, Santa Cruz Biotechnology, Dallas, TX, USA). For blood group antibodies, we used anti-K (#8000455463, Sanquin Reagents B.V., Amsterdam, Netherlands), anti-Fyb (#MFybM261-2, CE-Immundiagnostika GmbH, Neckargemünd, Germany), and anti-Jka (#OjkaM226-1, CE-Immundiagnostika GmbH). Puromycin (#110522221201, Beyotime Biotechnology, Shanghai, China) and 4% paraformaldehyde (#BL-539A, Biosharp, Hefei, China) were also purchased.

**CD47^high^293T cells and lentivirus infection:** Lentivirus overexpressing CD47 (#221021LVG23, Starfish organism) was prepared using EGFP and Puro(R), containing vectors with pLV[Exp]-EGFP:T2A:Puro-CMV>hCD47[NM_001777.4]. Lentivirus (multiplicity of infection = 30) was co-cultured with the HEK-293T cells. After 48 h, EGFP was observed using a laser confocal microscope and flow analysis to determine whether viral infection of the cells was successful. Puromycin (1.8 µg/mL) was used for screening CD47 cells with viral infections. Cells with Puro(R) were alive, whereas cells in the control group died. The living cells were cultured in Dulbecco’s modified Eagle’s medium supplemented with 10% fetal bovine serum and puromycin to obtain CD47^high^293T cells.

**Pre-transfusion testing:** For titration of free anti-CD47, patient plasma samples were serially diluted in saline until the RBCs no longer agglutinated. The anti-CD47 titers of patients’ plasma were determined using the diluted samples. For cross-matching, gel columns (filled with anti-globulin), saline, and polybrenes were used according to the manufacturer’s instructions or standard protocol of the AABB technical manual. Screening for irregular antibodies and reverse blood group typing were also performed using standard procedures, according to the AABB manual (20th edition). The agglutination reaction strength was scored by two immunohematology technicians blinded to the experiments on a scale of 0 (negative) to 4+ (strongest positive).

**Adsorption of anti-CD47mAb onto cells:** Cells (1 × 10^6^) were mixed with 200 μL plasma and incubated for 20 min at 37 ℃. The cells were removed by centrifugation at 1000 × *g* for 5 min, and the supernatant was collected for pre-transfusion testing.

**Flow cytometry analysis:** Flow cytometry analysis was performed to detect CD47 expression on cells. Briefly, 1 × 10^5^ cells were stained with PE-anti-human CD47 for 20 min in the dark at 4 °C. The cells were washed and resuspended for analysis (BD FACS Canto II, BD Biosciences, Franklin Lakes, NJ, USA).

**Western blot analysis:** The cells were lysed in radioimmunoprecipitation assay buffer (#P0013C, Beyotime). Proteins from different cells were separated using sodium dodecyl sulfate-polyacrylamide gel electrophoresis and transferred onto polyvinylidene fluoride membranes. The membranes were incubated overnight with anti-CD47 and anti-α-tubulin and then incubated with appropriate secondary horseradish peroxidase-conjugated antibodies. Blots were detected using a chemiluminescence detection kit (#34077, Thermo Fisher Scientific, Waltham, MA, USA).

**Statistical analysis:** All experiments were repeated at least five times. Agglutination was scored on a scale of 0 (negative) to 4+ (strongest positive). GraphPad Prism 8.4.2 software (GraphPad, Inc., La Jolla, CA, USA) was used for statistical analysis. Two groups were analyzed using the Student’s *t*-test. *P* <0.05 was set as the threshold of significance.

**Supplemental figures legends**

**S-Figure 1. Establishment of an interference model for pre-transfusion testing**

An interference model for pre-transfusion testing was established by adding anti-CD47mAb to normal plasma. (A) Interference was detected using the gel column method, and agglutination was evaluated in a concentration-dependent manner. (B) Interference in cross-matching was detected at a concentration of 200 ng/mL anti-CD47mAb using the saline and polybrene saline methods. (C) Interference in irregular antibody screening was observed upon administration of 200 ng/mL anti-CD47mAb. (D) Interference also occurred in the reverse B blood group typing using the saline tube method. Agglutination was scored on a scale of 0–4+.

**S-Figure 2. HEL cells partially depleted plasma anti-CD47mAb**

(A) Diagram of anti-CD47mAb depletion by HEL cells in plasma. HEL cells were incubated with plasma containing anti-CD47 and then removed *via* centrifugation. (B) Anti-CD47mAb (4 or 5 ng/mL) was added to the plasma to simulate interference. Agglutination decreased from 3+/2+ to 0 when 1.0 × 10^6^ HEL cells were used. (C) Anti-CD47mAb (10 or 20 ng/mL) was added to the plasma to model interference. Agglutination was detected before and after treatment with 1.0 × 10^6^ HEL cells, and partial depletion was observed. (D) Anti-CD47mAb (20 ng/mL) was added to the plasma to model interference. Agglutination was detected before and after treatment with 3.0 × 10^6^ HEL cells and scored on a scale of 0–3+.

**S-Figure 3. High CD47 expression was established in an HEK-293T cell line**

(A) Detection of EGFP in HEK-293T cells under a laser confocal microscope. After infection and screening, high levels of EGFP fluorescence were observed on 293T cells. Scale bar = 50 μm. (B) EGFP flow analysis also showed that 293T cells were infected with lentivirus carrying the CD47 coding sequence. (C) After lentiviral infection, 293T cells were stained with PE-anti-human CD47, and CD47 expression on the cells was detected using flow cytometry. CD47^high^293T cells were significantly increased. (D) Western blot analysis of CD47 from 293T cells before and after lentiviral infection showed that CD47 protein levels were increased in 293T cells.

**S-Figure 4. Fixation of CD47^high^293T cells depleted plasma anti-CD47mAb**

CD47^high^ 293T cells were fixed with 4% paraformaldehyde and stored at 4 °C. After storage for 1 or 2 weeks, CD47^high^293T cells were used to deplete anti-CD47mAb. (A) Agglutination in cross-matching using the gel column method decreased from 4+ to 0. (B) Agglutination in saline and polybrene-mediated cross-matching decreased from 3+/4+ to 0. (C) Agglutination in irregular antibody screening detected using the gel column method showed that interference was eliminated. (D) Abnormal agglutination in blood group B reverse typing was also depleted. (E) After storage for 3 months, CD47^high^293T cells were used to deplete anti-CD47mAb. Agglutination in cross-matching using the gel column method decreased from 4+ to 1+. Agglutination was scored on a scale of 0–4+.
