# Supplementary material for: Depletion of anti-CD47mAb in plasma by genetically modified cells for pre-transfusion testing
Source: Genes Dis. 2023 Sep 13;11(5):101104. doi: 10.1016/j.gendis.2023.101104 (PMC11176643; doi:10.1016/j.gendis.2023.101104)
Supplement: Multimedia component — 2 [file mmc2.pptx]

## Slide 1
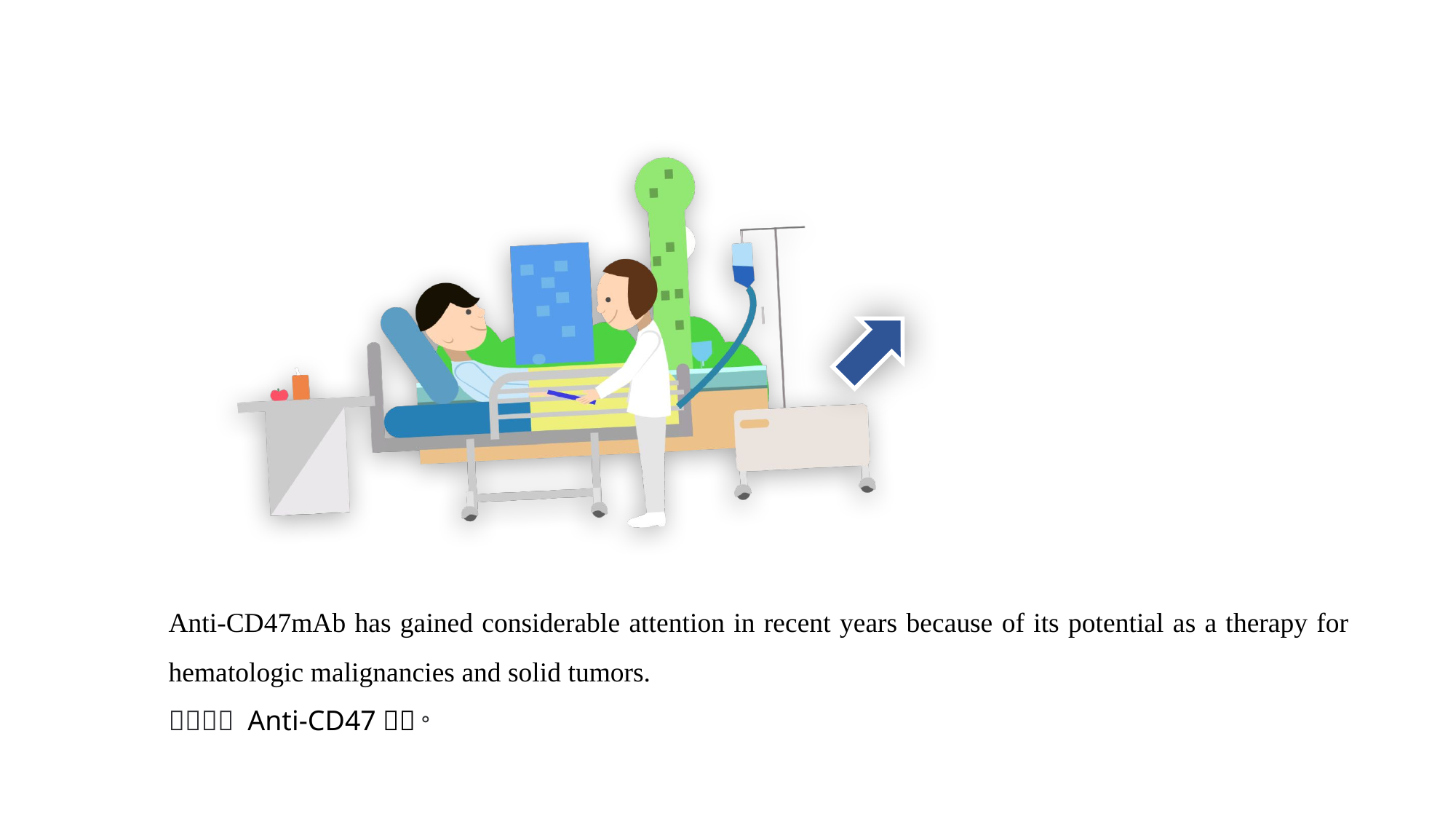

Anti-CD47mAb has gained considerable attention in recent years because of its potential as a therapy for hematologic malignancies and solid tumors.
近年来， Anti-CD47单抗作为血液恶性肿瘤和实体肿瘤的潜在治疗药物，引起了全球广泛关注。

## Slide 2
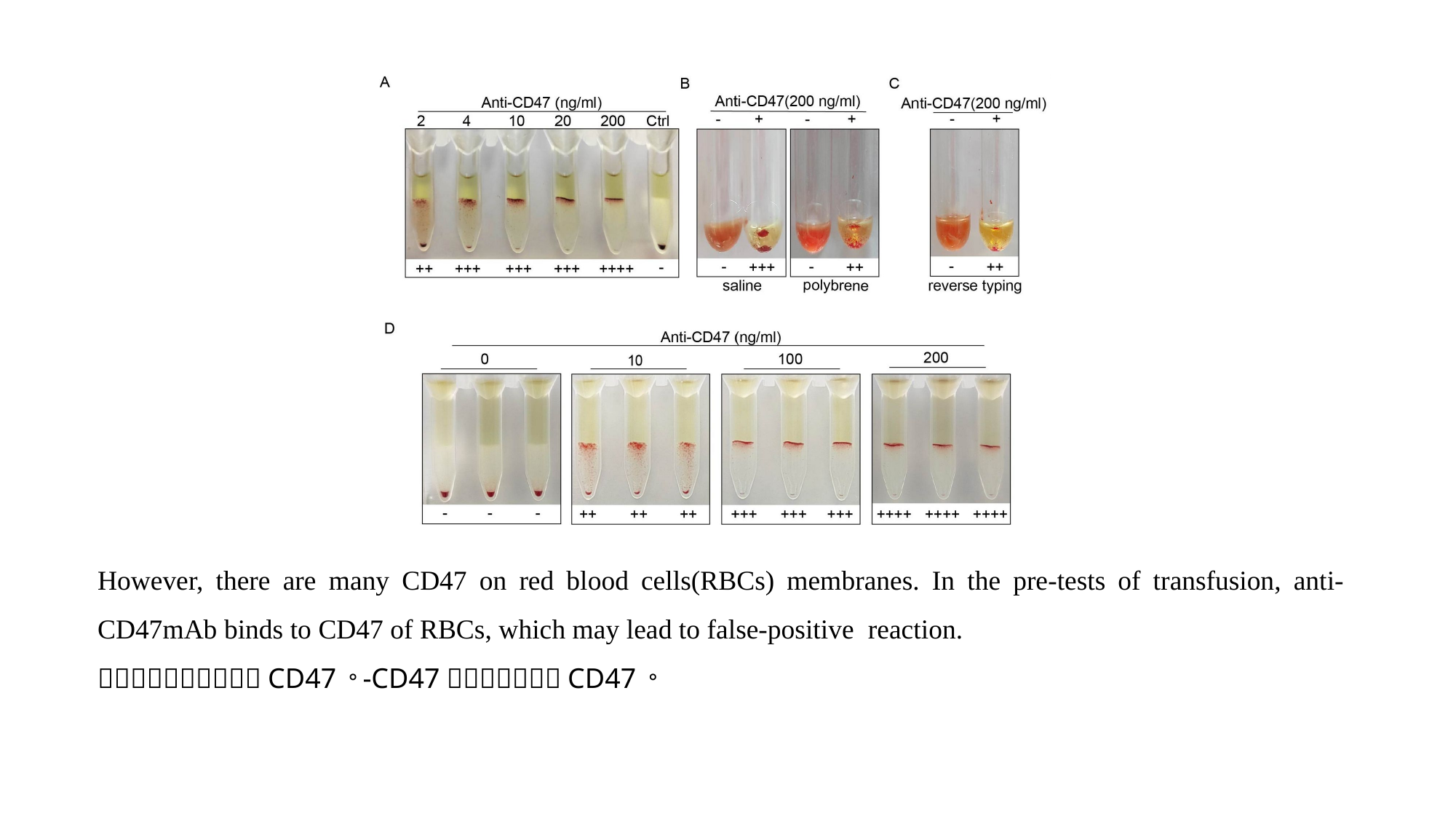

However, there are many CD47 on red blood cells(RBCs) membranes. In the pre-tests of transfusion, anti-CD47mAb binds to CD47 of RBCs, which may lead to false-positive reaction.
然而红细胞膜上有许多CD47。在输血前检测中，抗-CD47单抗与红细胞的CD47结合，可能导致假阳性反应。

## Slide 3
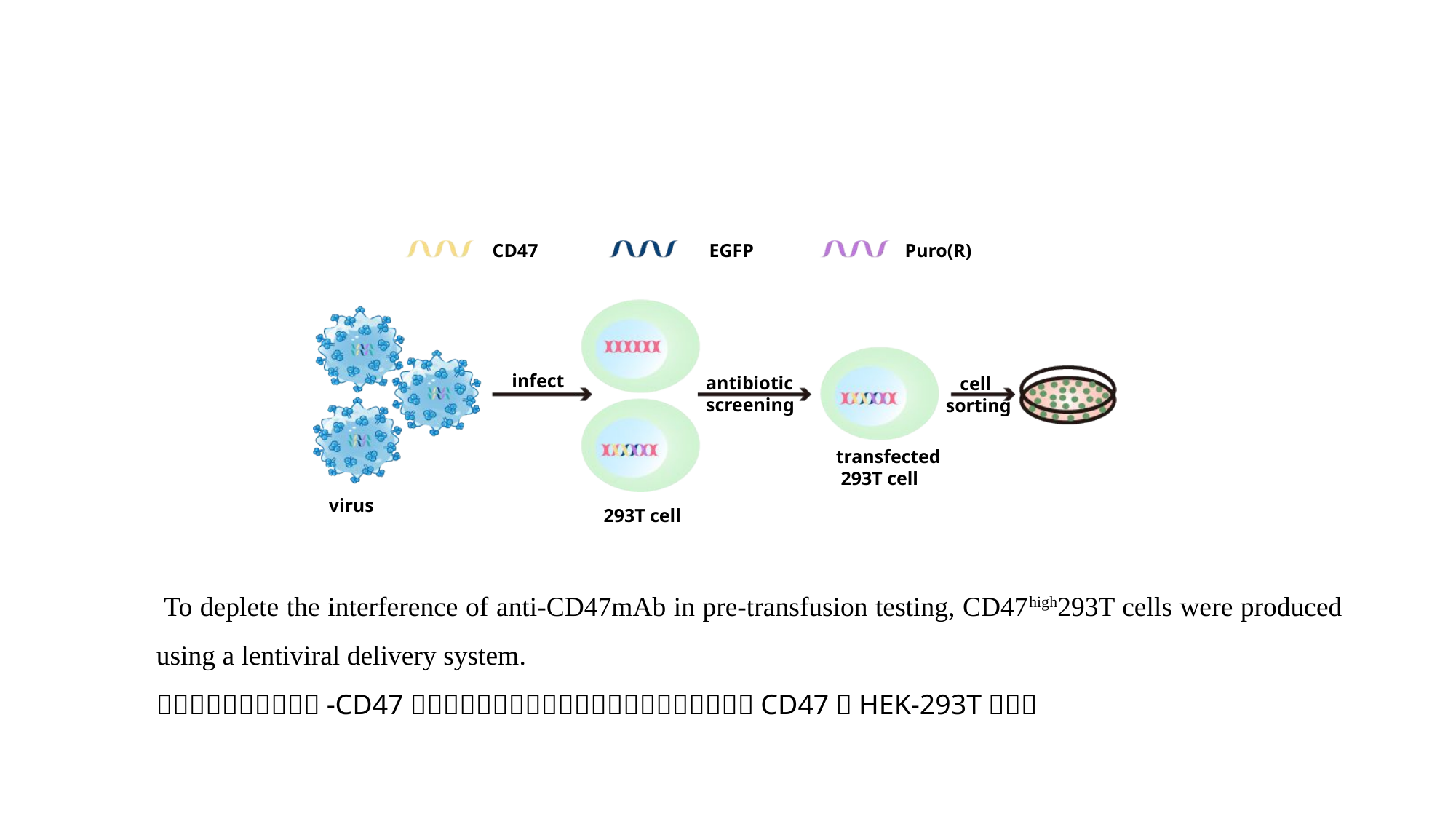

EGFP
CD47
Puro(R)
infect
antibiotic
screening
 cell
sorting
transfected
 293T cell
virus
293T cell
 To deplete the interference of anti-CD47mAb in pre-transfusion testing, CD47high293T cells were produced using a lentiviral delivery system.
为消除输血前检测中抗-CD47单抗的干扰，使用慢病毒系统表达制备了高表达CD47的HEK-293T细胞。

## Slide 4
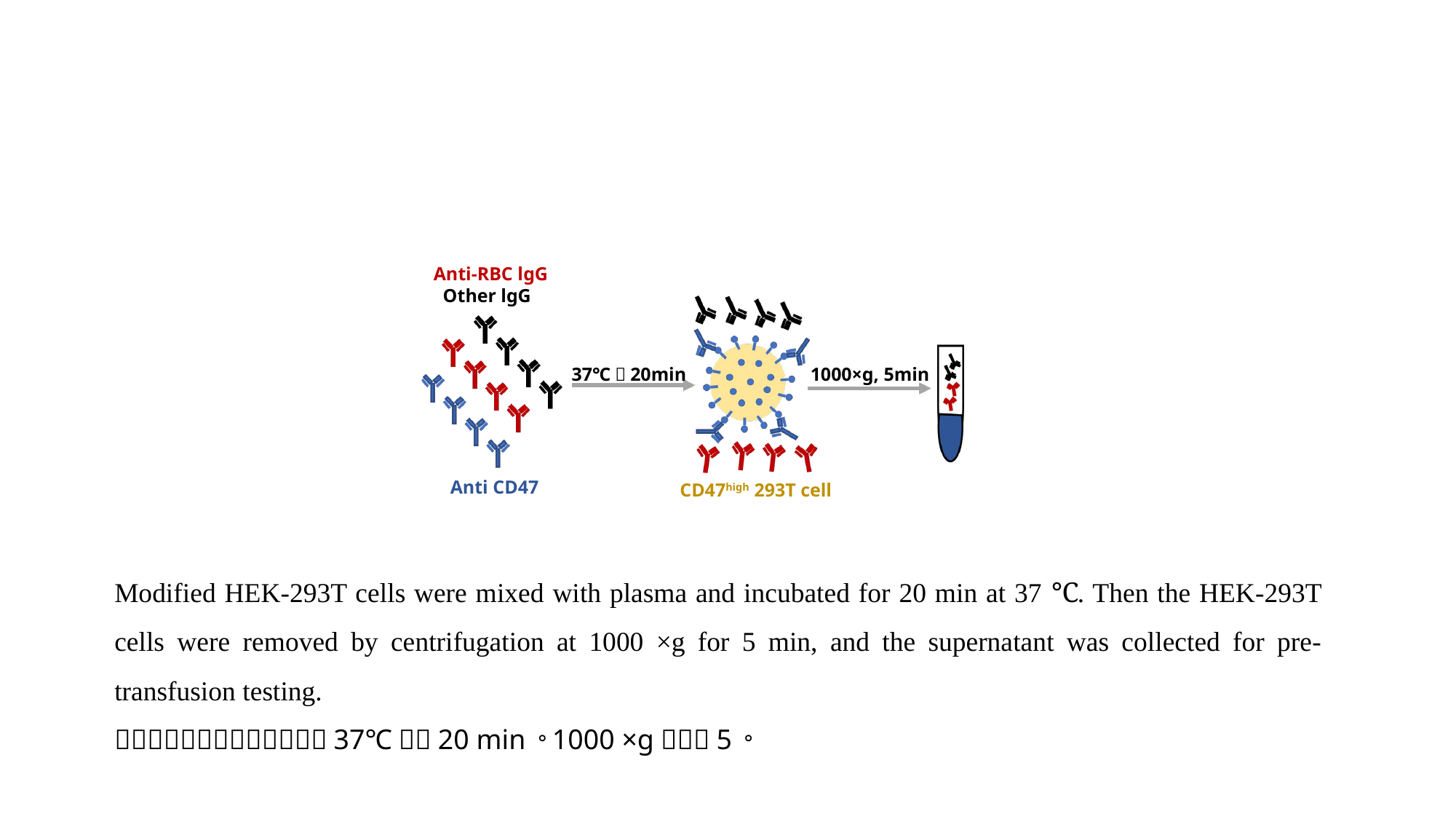

Anti-RBC lgG
 Other lgG
37℃，20min
1000×g, 5min
Anti CD47
CD47high 293T cell
Modified HEK-293T cells were mixed with plasma and incubated for 20 min at 37 ℃. Then the HEK-293T cells were removed by centrifugation at 1000 ×g for 5 min, and the supernatant was collected for pre-transfusion testing.
将过表达的细胞与血浆混合，37℃孵育20 min。在1000 ×g下离心5分钟，取出细胞，收集上清进行输血前检测。

## Slide 5
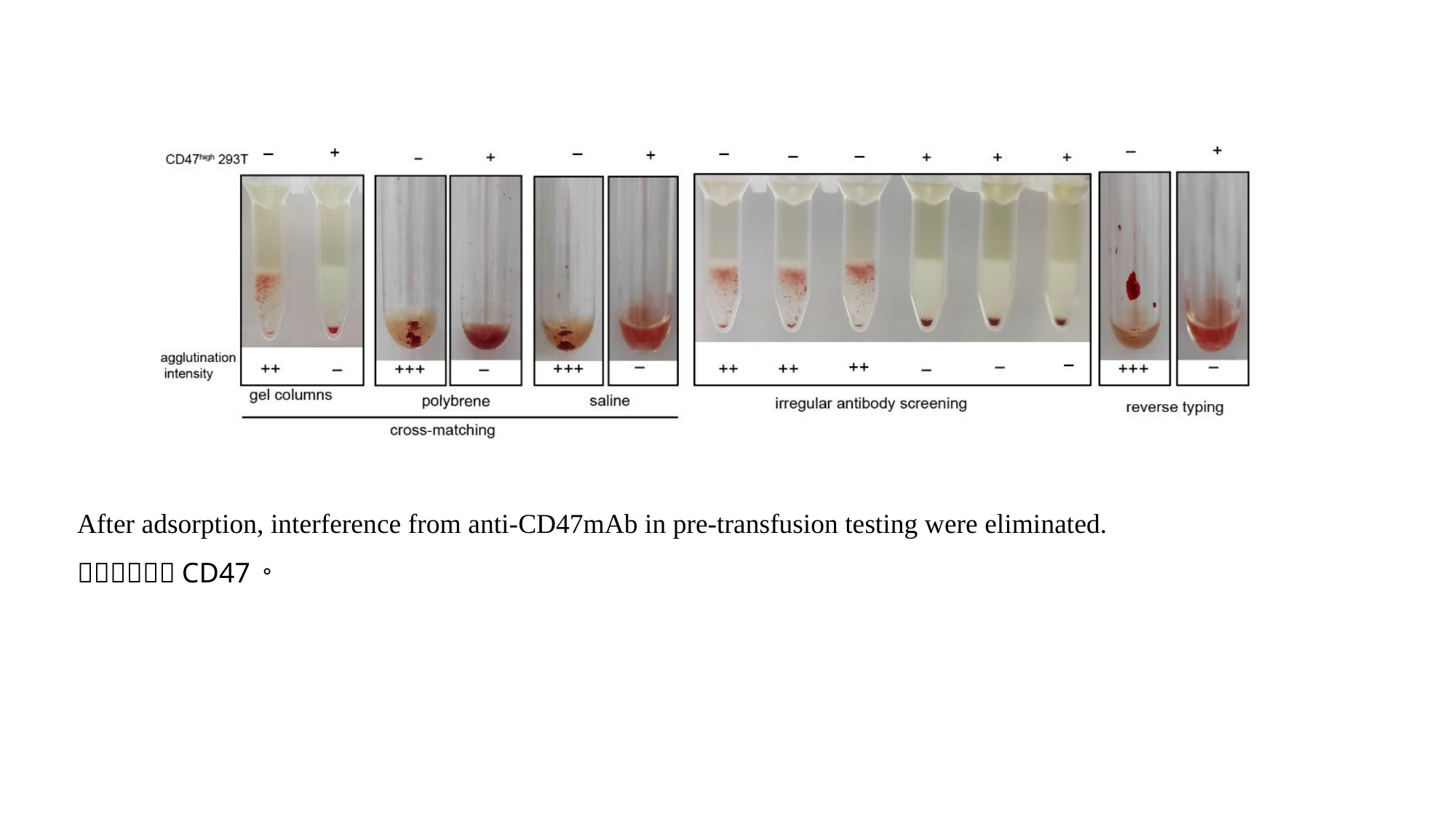

After adsorption, interference from anti-CD47mAb in pre-transfusion testing were eliminated.
吸附后消除了CD47单抗对输血前检测的干扰。
